# Optimized analysis of blood flow and wall shear stress in the common carotid artery of rat model by phase-contrast MRI

**DOI:** 10.1038/s41598-017-05606-4

**Published:** 2017-07-12

**Authors:** Shin-Lei Peng, Cheng-Ting Shih, Chiun-Wei Huang, Shao-Chieh Chiu, Wu-Chung Shen

**Affiliations:** 1 Department of Biomedical Imaging and Radiological Science, China Medical University, Taichung, Taiwan; 2 3D Printing Medical Research Center, China Medical University Hospital, China Medical University, Taichung, Taiwan; 3 Center for Advanced Molecular Imaging and Translation, Chang Gung Memorial Hospital, Taoyuan, Taiwan; 4 Department of Radiology, China Medical University Hospital, Taichung, Taiwan

## Abstract

The present study systemically investigated the influence of gated/non-gated sequences, velocity encoding (VENC), and spatial resolution on blood flow, wall shear stress (WSS), and artery area evaluations when scanning the common carotid artery (CCA) in rats using phase-contrast magnetic resonance imaging (PC-MRI). We first tested whether or not non-gated PC-MRI was appropriate for evaluating blood flow and WSS in rats. For both gated and non-gated techniques, VENC values in the range of 60–120 cm/s with an interval of 10 cm/s were also tested. Second, we optimized the in-plane resolution of PC-MRI for blood flow and WSS measurements. Results showed the usage of a gated instrument can provide more reproducible assessments, whereas VENC had an insignificant influence on all hemodynamic measurements (all *P* > 0.05). Lower resolutions, such as 0.63 mm, led to significant overestimations in blood flow and artery area quantifications and to an underestimation in WSS measurements (all *P* < 0.05). However, a higher resolution of 0.16 mm slightly increased measurement variation. As a tradeoff between accuracy and scan time, we propose a gated PC-MRI sequence with a VENC of 120 cm/s and a resolution of 0.21 mm to be used to extract hemodynamic information about rat CCA.

## Introduction

Evaluating local flow profiles in vessels is a useful means to assess cardiovascular function and physiopathological conditions^1, 2^. It is expected to gain increased popularity in both clinical routine and pre-clinical studies. Doppler ultrasound (US) and phase-contrast (PC) MRI are the methods of choice for measuring flow-related parameters. Doppler US has the advantage of better temporal resolution to capture the peak systolic blood flow velocity. However, US measurement requires considerable technical ability, and it is therefore susceptible to the operator dependency^3^. By using phase shifts in moving spins to quantify the velocity information in flowing vessels, PC-MRI enables the noninvasive assessment of blood rheology^4, 5^. Because it is user-independent and allows straightforward flow quantification, PC-MRI has had an immediate impact on various fields.

The major use of PC-MRI is to provide insight into the hemodynamic characteristics of blood flow in large vessels, such as cerebral vessels and carotid arteries. By measuring and integrating flow volume from the main feeding arteries of the brain, e.g., the left/right internal carotid arteries (ICA) and the vertebral artery (VA), one can quantify whole brain cerebral blood flow (CBF)^6, 7^. Wall shear stress (WSS) is the frictional force exerted on the endothelium of vessel walls by the circulation. Prior studies have shown that common carotid arteries (CCA) with a relatively low WSS are prone to developing atherosclerosis^8^ and are associated with other cerebrovascular diseases^9^. PC-MRI covers a wide range of significant applications and the derived parameters allow comparisons between normal and pathological blood flow. Optimizing the scanning parameters for PC-MRI is therefore nontrivial, and they have been assessed in many human studies^10–17^.

Pre-clinical animal models, such as rats, are essential steps toward better understanding of cardiovascular disease and also have considerable clinical implications. Therefore, an increasing number of studies on hypertension^18^ and stenosis^19^ use PC-MRI to reveal flow-relative information in animal models. Reports on optimizing the scanning parameters for PC-MRI in rat models are scarce, however. This may hamper the utilization of this technique in a broad scope of applications.

Prior human studies have revealed that blood flow measurements using the PC-MRI technique were not affected by using electrocardiogram (ECG)-gated implementations^13^. Taking considerable difference in scan duration into account, non-gated PC-MRI has gained increasing popularity in human PC-MRI studies^20–22^. For rat PC-MRI studies, however, vessel diameters are relatively small, and their hemodynamic features are significantly different when compared to those of humans. Therefore, the central goal of this study is to test whether non-gated PC-MRI is appropriate for rat PC-MRI examinations for not. Moreover, the selection of velocity encoding (VENC) and spatial resolution settings are linked to precise quantifications. A lower VENC value benefits the velocity-to-noise ratio (VNR) and improves the image quality^23^. However, an underestimated VENC parameter introduces phase aliasing and necessitates extra corrections^24^. On the other hand, higher spatial resolution is expected to provide a more accurate measurement but a potential issue is the longer scan duration^16^. Given that accuracy and time efficiency are trade-offs, it is desirable to establish optimized scanning parameters for PC-MRI for the accurate quantification of hemodynamic characteristics in rats.

In this study, we examined the influence of gated/non-gated sequences, VENC, and spatial resolution on blood flow, WSS, and artery area measurements, aiming at proposing optimized parameters for PC-MRI when scanning CCA in rats. CCA in rat is an excellent model for studying hypertension^25, 26^, stenosis^26^, and stroke^27^. Especially, CCA is the predilection for the development of atherosclerotic plaques, and low WSS values contribute to the initiation and progression of atherosclerotic disease^28^. Investigation of WSS in CCA in rat models could provide more comprehensive information. Results from this study may fill the gap between theory and preclinical animal studies.

## Results

### Study 1

**Figure 1:**
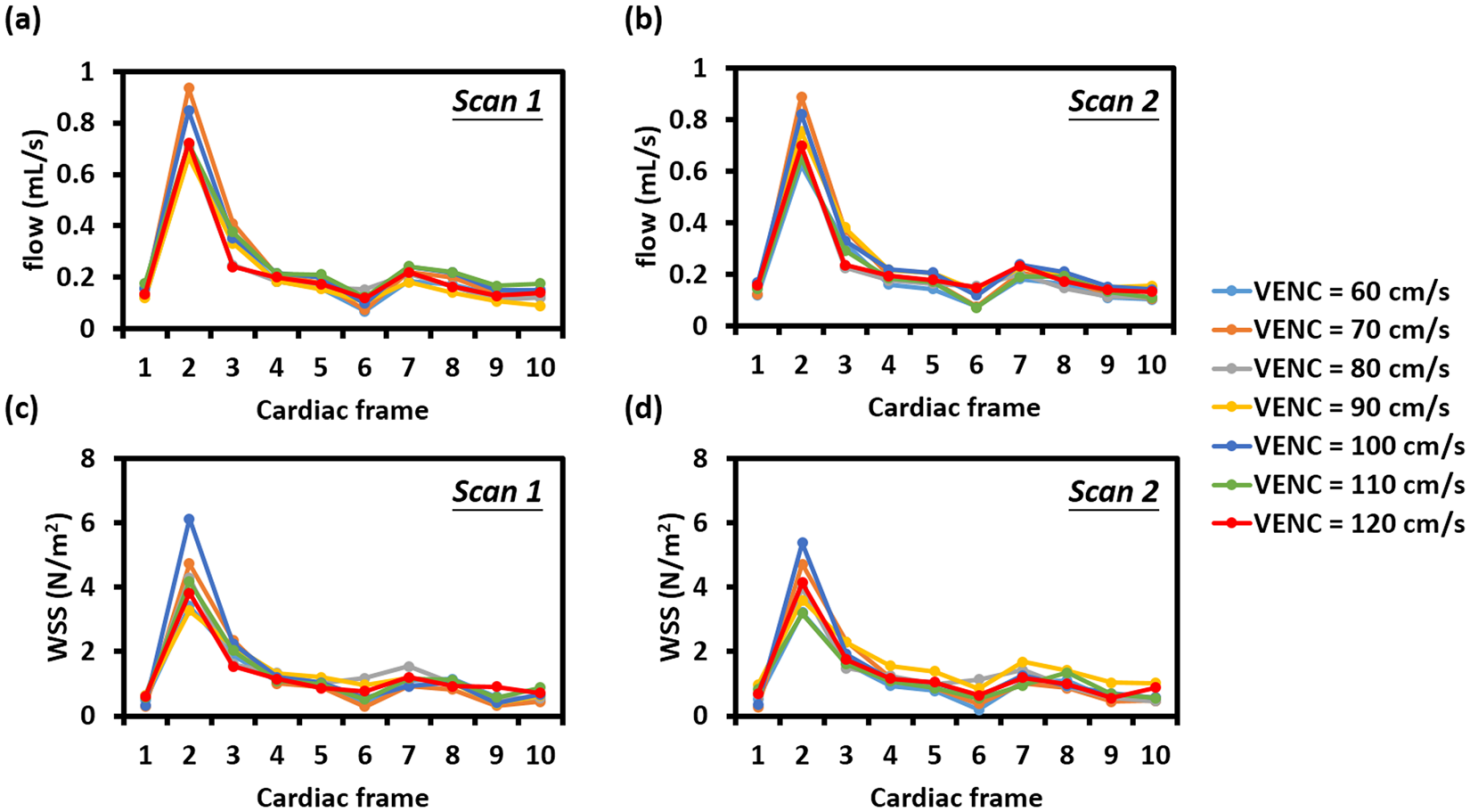
One representative set of time-resolved profiles from different VENC settings for blood flow from (**a**) scan 1 and (**b**) scan 2 and WSS from (**c**) scan 1 and (**d**) scan 2 by the gated PC-MRI strategy. Both blood flow and WSS show repeatable measurements.

In Study 1, gated/non-gated techniques with varied VENC settings were tested. Figure 1 shows one representative set of time-resolved profiles from different VENC settings for blood flow (Fig. 1a and b) and WSS (Fig. 1c and d) in the rat CCA recorded by the gated PC-MRI strategy. To demonstrate test–retest reproducibility, scans were made twice for each VENC setting without repositioning. Generally speaking, both blood flow and WSS show repeatable measurements.

**Figure 2:**
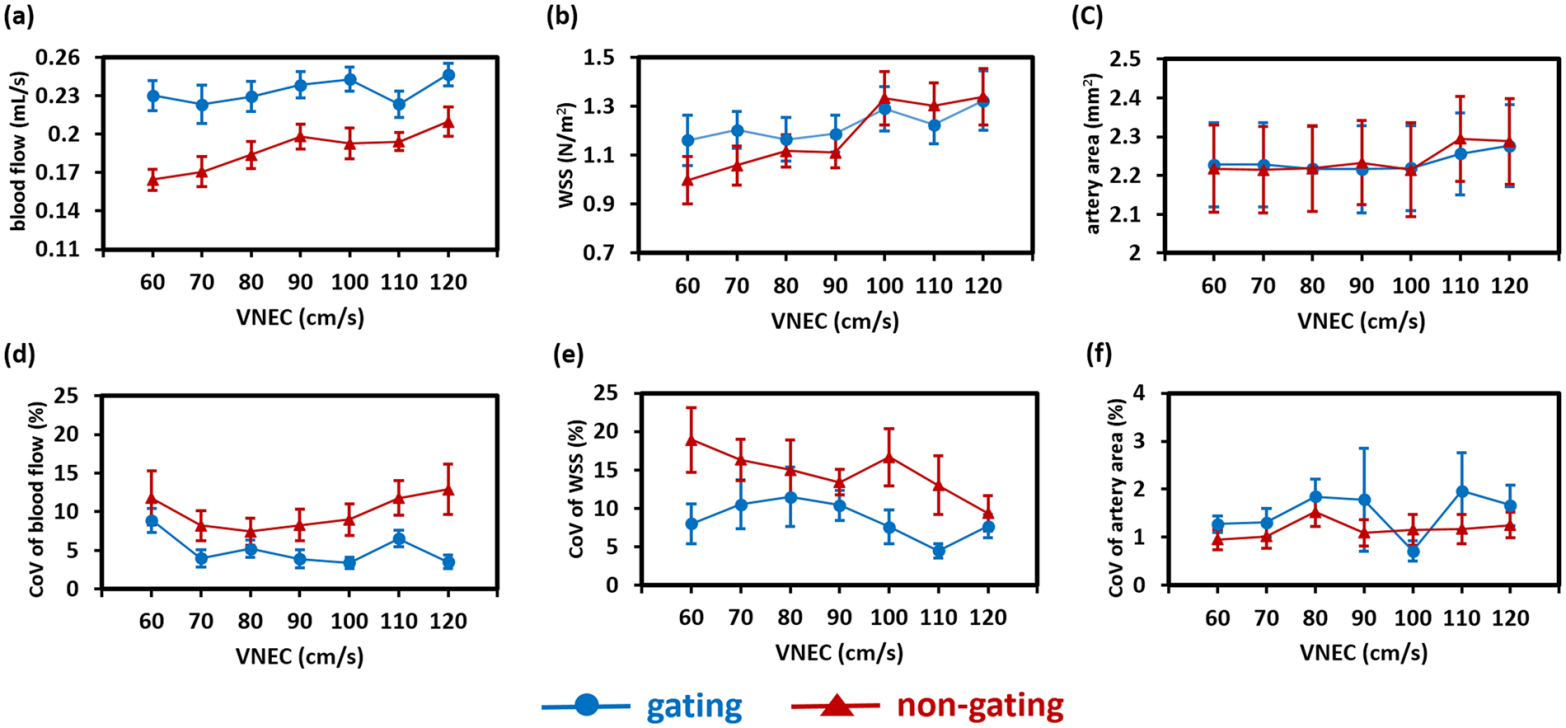
Influences of gated and non-gated strategies and varied values of VENC on (**a**) blood flow, (**b**) WSS, (**c**) artery area, and their corresponding CoVs ((**d**), (**e**), and (**f**), respectively).

Results for blood flow, WSS, artery area, and corresponding coefficients of variation (CoVs) from gated/non-gated strategies and varied values of VENC are displayed in Fig. 2. Two-way analysis of variance (ANOVA) with repeated measurement shows that the usage of a gated sequence has a significant impact on flow (Fig. 2a, *P* < 0.001), but not on WSS (Fig. 2b, *P* = 0.39) and artery area (Fig. 2c, *P* = 0.92). On average, the blood flow measured with non-gated PC-MRI was significantly lower than that with the gated technique. The average differences between gated and non-gated measurements ranged from 13.06% to 28.53% for different VENC settings (*P* < 0.05 for all VENC settings). Moreover, the gated sequence demonstrates smaller CoV in blood flow (Fig. 2d, *P* < 0.001) and WSS (Fig. 2e, *P* < 0.001) when compared to those from the non-gated one, suggesting the gated sequence has an advantage in intrascan reproducibility. In terms of artery size measurement (Fig. 2e), the CoV between gated and non-gated strategies was not significant (*P* = 0.16).

With the help of the gated sequence, the values of blood flow, WSS, artery area, and their corresponding CoV values were independent of the settings of VENC (one-way ANOVA with repeated measurement, all *P* > 0.05), suggesting gated sequence provides a higher tolerance level for different VENC settings. For the non-gated sequence, on the other hand, the VENC setting had a significant impact on flow measurement (*P* < 0.001). The blood flows were 0.16 ± 0.008 and 0.21 ± 0.01 mL/s for VENC of 60 cm/s and 120 cm/s, respectively. Post-hoc Tukey’s honest significant difference (HSD) test showed that the blood flow value obtained with a VENC of 60 cm/s was significantly underestimated when compared to that obtained with a VENC of 120 cm/s (*P* < 0.05).

Although a gated sequence sacrifices scan time, it provides more reliable measurements, and the obtained values are not confounded by varying the VENC settings. We therefore employed a gated sequence with a VENC of 120 cm/s for the second part of our study (Study 2). Under these circumstances, no phase-wrapping correction was needed in the following section.

### Study 2

**Figure 3:**
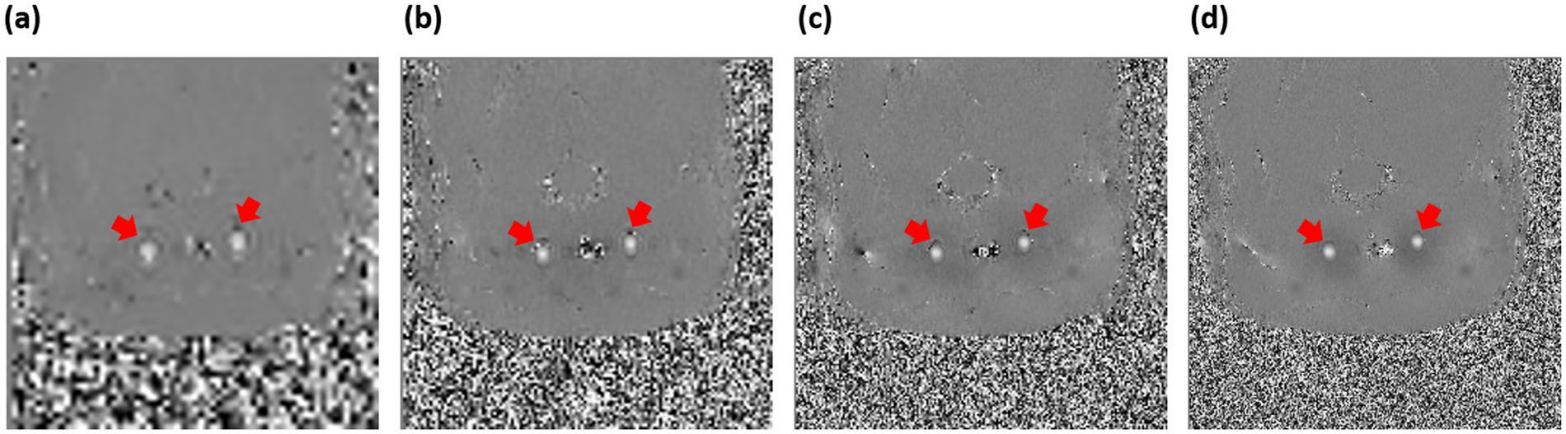
Phase images from a representative rat CCA with in-plane resolution of (**a**) 0.63 mm, (**b**) 0.31 mm, (**c**) 0.21 mm, and (**d**) 0.16 mm. Red arrows indicate the locations of CCA.

In Study 2, flow-derived parameters measured with gated PC-MRI sequences at different spatial resolutions were compared. Phase images with different values of in-plane resolution from a representative rat CCA are shown in Fig. 3. When the resolution was as low as 0.63 mm, it was clear that the artery was ovoid in shape, suggesting a higher degree of partial volume effects. In contrast, the boundary of the artery became round and more resolved when a higher resolution, such as 0.21 mm, was employed.

**Figure 4:**
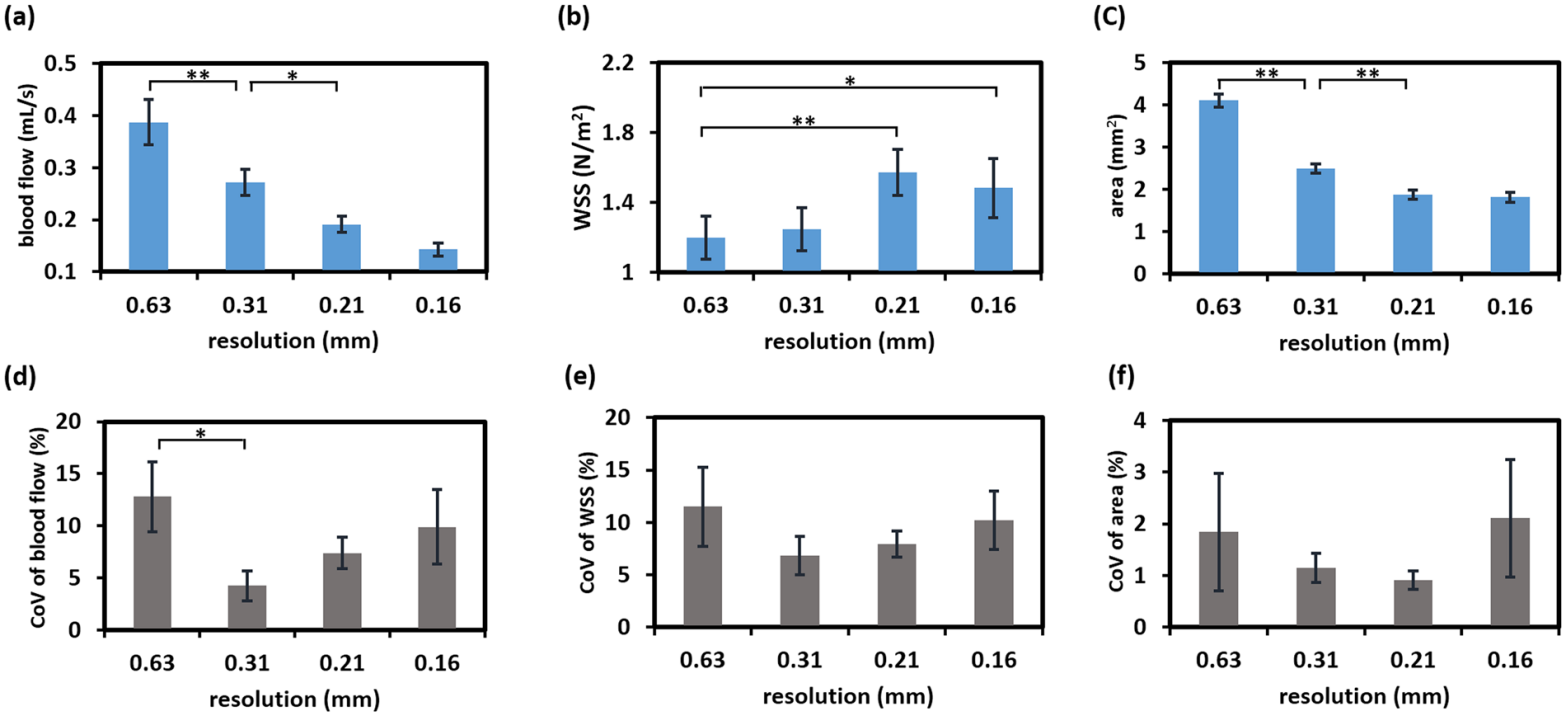
Effects of different in-plane resolution values on (**a**) blood flow, (**b**) WSS, (**c**) artery area, and their corresponding CoVs [(**d**),(**e**) and (**f**), respectively]. *P < 0.05, **P < 0.01.

The measured blood flow, WSS, artery area, and their corresponding CoVs for rat CCA as a function of in-plane resolution are shown in Fig. 4. As expected, spatial resolution considerably influenced all parameters. One-way ANOVA with repeated measurement shows that in-plane resolution plays a significant role in blood flow (Fig. 4a, *P* < 0.001) and artery area (Fig. 4c, *P* < 0.001). A post-hoc Tukey’s HSD test revealed that the lower resolutions, such as 0.63 mm and 0.31 mm, led to an overestimation of the blood flow and artery area due to the partial volume effect. Resolutions of 0.63 mm and 0.31 mm significantly overestimated blood flow by 171.4% (*P* < 0.01) and 90.1% (*P* < 0.01), respectively, when compared to that obtained with the finest resolution of 0.16 mm. But when the resolution was 0.21 mm, the resulting blood flow and artery area were comparable to those obtained with the finest resolution of 0.16 mm (both *P* > 0.05).

WSS measured by gated PC-MRI was found to be significantly dependent on the in-plane resolution (Fig. 4b, *P* < 0.05) as well. At the limited resolution of 0.63 mm, the obtained WSS was significantly lower than that obtained from the finest resolution of 0.16 mm (*P* < 0.05). No significant difference in WSS was found when the resolution was 0.31 mm or higher (*P* > 0.05 for all pairwise comparisons).

Interestingly, the CoVs of blood flow, WSS, and artery area tended to be slightly greater when both the lowest resolution of 0.63 mm and the highest resolution of 0.16 mm were employed. One-way ANOVA with repeated measurement further revealed that spatial resolution has a significant impact on CoV of blood flow (Fig. 4d, *P* = 0.03), but not on those of WSS (Fig. 4e, *P* = 0.44) and artery area (Fig. 4f, *P* = 0.6). With the resolution of 0.63 mm, the intrascan CoV of blood flow was significantly larger than that with the resolution of 0.31 mm (*P* < 0.05).

The summarized *P* values of the hemodynamic measurements from both Study 1 and Study 2 were tabulated in Table 1.

**Table 1:** The summary of *P* values of the hemodynamic measurements from both Study 1 and Study 2.

	Gated/non-gated strategies	VENC setting (for gated strategy only)	Spatial resolution
Flow (mL/s)	<0.001	0.21	<0.001
WSS (N/m^2^)	0.39	0.42	<0.05
Artery area (mm^2^)	0.92	0.38	<0.001
CoV of flow	<0.001	0.14	<0.05
CoV of WSS	<0.001	0.45	0.44
CoV of artery area	0.16	0.76	0.6

## Discussion

In this work, the influence of gated/non-gated sequences, VENC, and in-plane resolution on the hemodynamic characteristics of rat CCA obtained using PC-MRI were examined. Two systematic studies were conducted to shed more light on the importance of the set scanning parameters. We first showed that the usage of cardiac-gated implementations in PC-MRI can provide more reproducible results; on the other hand, when including ECG gating, the derived blood flow, WSS, and artery area were not affected by the VENC settings. Additionally, we explored the effect of in-plane resolution on quantifying blood flow, WSS, and artery area, showing that all parameters vary as a function of spatial resolution. To reach a compromise for the tradeoff between accuracy, test-retest reproducibility, and time consumption, the resolution of 0.21 mm with gated PC-MRI sequences is suggested for extracting hemodynamic information about rat CCA. The present study intends to fill the gaps between theory and pre-clinical applications.

The non-gated PC-MRI sequence, because of its short scan time, allows measurements of the blood flow volume in a variety of human studies^20, 21^. The accuracy and reproducibility of non-gated PC-MRI in terms of blood flow measurement have been discussed well and are supported by some studies^12, 13^, but others have raised questions about them^14^. Therefore, the ongoing discussion in this work is whether the flow-related parameters in the rat model should be measured using a cardiac-gated or a non-gated PC-MRI technique. Our results show that, although the scan time is dramatically reduced when a non-gated PC-MRI is employed, gated PC-MRI significantly improves the reproducibility in both blood flow and WSS measurements. Moreover, the flow measured with the non-gated PC-MRI was significantly lower than that obtained with the gated sequence. One possible reason is that the heart rates of rats are as fast as 400 bpm, even under anesthesia with 2% isoflurane. The rapid alternation between systolic and diastolic phases increases the signal fluctuations, reducing the fidelity of the data. Therefore, averaging flow information over the cardiac cycle may give rise to a loss of exact information during the systolic phase, resulting in artificially lower flow values. Indeed, we did find that the mean velocity within the artery ROI was significantly small for the non-gated method (*P* < 0.05, see Supplementary Fig. S1), suggesting that the peak velocity was lost during the data collection. Since accurate measurement is on the list of priority, the gated PC-MRI is recommended for flow measurement in rat models. Non-gated PC-MRI may only serve as a rough estimation.

Temporal resolution also plays a significant role in a trade-off between the accuracy of hemodynamic parameter measurements and the scanning duration. Early work done by Cibis *et al*.^17^ has shown that peak velocity had an inverse relationship with temporal resolution, by showing that lower temporal resolution tends to underestimate the peak velocity. If the temporal resolution was as high as 24.4 ms, the measured results were similar to the one measured by the ultrasound probe. In the present study, the temporal resolution for the gated sequence was around 31 ms, which should be sufficient to capture the flow change. Interestingly, in the same study, they reported that the WSS values averaged over the cardiac cycle were independent of the temporal resolution, but the cause of this phenomenon remains to be elucidated. To some extent, the gated sequence with a longer temporal resolution could be considered as the non-gated sequence. Moreover, the gating effect on WSS have not examined before. In this study, we did find that the non-gated sequence significantly underestimated the mean velocity and flow values, but not the WSS. The shear tensor is proportional to the flow velocity gradient. Even though the non-gated method significantly underestimates the flow velocity within the artery ROI, the relative change in the velocity profile remains similar (see Supplementary Fig. S2). The unaffected WSS values are thus expected. Our results were in good agreement with their findings. Therefore, if the WSS is the only hemodynamic parameter of interest, the measurement from the non-gated method could be taken into consideration.

In the PC-MRI settings, determination of the VENC value is critical. The recommendation is to choose a VENC value that is adequate for eliminating the unnecessary phase aliasing while simultaneously minimizing the velocity noise in the measured flow field. The effect of VENC on WSS measurements has been described well in the literature. Both simulations^15^ and human studies^10^ have proved that the VENC setting has an insignificant impact on the WSS estimates. Observations in the present study are in good agreement with these prior findings. However, the effect of VENC settings on the flow measurement has not been fully discussed. In a standard PC-MRI experiment, a single VENC value higher than the peak velocity is set to avoid aliasing. However, higher VENC is confounded by a worse VNR^23^, which increases the noise in the lower velocity environment and leads to inaccurate measurements. A multiple VENC approach has been proposed to improve the VNR by tailoring the VENC settings according to the systolic or diastolic cardiac phases^29, 30^. The low-velocity flow components can be determined precisely and thus quantification of the flow patterns can be improved. In Study 1, a mono VENC was used throughout the entire cardiac cycle for each VENC setting. To improve the VNR, we intentionally collected data with the number of excitations (NEX) of 8, and the resulting VNR was around 22 for VENC of 120 cm/s, which was comparable to that from multi-VENC techniques^31^. Consequently, the flow measurements were not biased by the VENC setting (Fig. 2a). Since a lower VENC introduces phase-aliased data and the phase-unwrapping technique requires additional time, we recommend a higher VENC with sufficient VNR as more feasible in the standard PC-MRI setting for rat models.

The PC-MRI parameters, such as spatial resolution, considerably influence all the flow-related parameter measurements. A limited spatial resolution is preferable for shorter scan times but significantly biases the quantification due to the partial volume effect. One study investigated the effect of resolution on quantifications of blood flow in the human ICA and VA^16^. The findings showed that, when the voxel size was extended by a factor of 2, the flow was overestimated by 5.5% and 13.3% for ICA and VA, respectively. The resolution effect was more pronounced in smaller vessels, such as the VA. In our Study 2 of rat CCA, when the voxel size was increased by a factor of 2, the blood flow was overestimated by 90.1%. Our findings are generally in line with the idea that smaller vessels are more vulnerable to the partial volume effect. Although higher in-plane resolution provides more accurate quantifications, it should be implemented with caution. Given that higher resolution is inversely proportional to the signal-to-noise ratio, the test–retest reproducibility, indicated by CoV, was slightly unstable when higher spatial resolution was used. Moreover, higher spatial resolution significantly lengthened the scan time. Taking into account the tradeoff between scan time, correction, and test–retest reproducibility, the resolution of 0.21 mm can be considered an optimized resolution for PC-MRI in rat CCA measurements.

The effect of spatial resolution on the magnitude of WSS has been discussed previously^11, 15, 17, 32^, and all suggested that the spatial resolution has the opposite effect on WSS estimation when compared to that on flow quantification. That is, WSS values were underestimated with lower spatial resolution. In this regard, the observation in the present study is in good agreement with these prior findings. For the underestimation in WSS values, the following possible mechanisms may be responsible. One is the inaccurate definition of vessel wall position. To calculate the WSS, the identification of vessel wall is on the list of priority^33^. While large voxels suggest spatial averaging, leading to the overestimations of lumen size and vessel diameter. Therefore, it is reasonable to expect that the velocity gradient is small. A second possible mechanism is that the peak velocity may be skewed and omitted as a result of partial volume effects^24^. This is an important issue for vessels whose diameters are less than 2–3 mm. However, the existing studies are mainly focused on phantom^17^ or numerical simulations^15^. The *in vivo* studies were restricted to a limited range of spatial resolution settings^11, 32^. To our knowledge, the dependence of WSS on varied spatial resolution values has never been tested in pre-clinical animal studies. Flow characteristics are far more complex *in vivo*, and physiological noise and cardiac pulsation may enhance the intricacies. In addition, the vessel sizes of rats are relatively small, which emphasizes the need for optimized scanning parameter settings. Here we suggest a resolution of 0.21 mm for future WSS measurements in the rat CCA model. A standardized resolution will allow measurements of WSS magnitudes across different centers to be compared. This could be important for the evaluation of atherosclerosis, which is characterized by lower WSS^28, 34^.

To compensate for the associated signal loss, the number of signal average (NEX) of 0.21 mm resolution was 12 and the resultant scanning time was around 9 minutes in this study. A straightforward alternative to increase signal-to-noise ratio (SNR) while reducing the scanning time would be to perform experiments at higher field strength^35^. Benefits of performing PC MRI at high fields include better visualization of blood flow and reduced velocity noise artifacts^36, 37^. However, when conductive fluid such as blood moves across a magnetic field, there is a voltage induced in the direction orthogonal to both the magnetic field lines and the direction of flow of the fluid^38, 39^. This phenomenon is so-called magneto-hydrodynamic (MHD) effect and proportional to the strength of magnetic field. The MHD effect can potentially cause the magnitude of the T-wave to become greater than that of the R-wave, leading to the faulty cardiac triggering. This phenomenon worsens the quality of cardiac gating at high magnetic fields. The effect of MHD in animal studies has never been discussed before. Further studies are suggested to discuss this potential influence in rat PC MRI studies at relatively high field.

In addition to WSS, the temporal oscillation of WSS during the cardiac cycle quantified by the oscillatory shear index (OSI) is another useful index to evaluate the initiation and progression of atherosclerotic disease. Several studies have proved the potential of PC-MRI in OSI estimations between pathophysiological and healthy conditions in human studies^40, 41^. The influence of spatiotemporal resolutions on OSI is vessel-dependent. Vessels with lower OSI tend to be more vulnerable to the spatial resolution, but not the temporal resolution^17^. Should there be the same scenario in rat studies, this might be a paramount direction for future works.

Although only CCA was evaluated in this work, this optimal protocol could be applied to other major arteries as well, such as the basilar artery. The basilar artery is one of the arteries that supplies the brain with oxygen. By measuring the flow change in the basilar artery during challenges such as CO_2_ inhalation and acetazolamide injection, cerebrovascular reactivity (CVR) could be quantified. CVR is a useful index to evaluate the brain perfusion reserve. Previous studies have employed the blood oxygenation level-dependent (BOLD)^42^ or arterial spin labeling (ASL)^43^ for CVR quantification. However, the BOLD MRI measurement relies primarily on the blood oxygenation level instead of pure perfusion information; the major disadvantage of ASL is the intrinsic low SNR, and this remains a challenge for animal studies. Although lacking spatial specificity, the PC-MRI provides a simple alternative for flow quantification, which could be used in preclinical applications to evaluate cerebral hemodynamics.

The findings from our study need to be interpreted in the context of its limitations. To begin, the information presented in this study was acquired by the 2D PC-MRI technique. Time-resolved multi-directional velocity-encoding 3D PC-MRI techniques could shed more light on the spatial aspects of the velocity distribution and flow structures. Optimizing the scanning parameters for 3D PC-MRI in the rat CCA model will be the focus of future studies. Second, in this study, only normal Sprague–Dawley (SD) rats were scanned. It has been shown that the vascular tone could be different in hypertensive rats^18^, and this may influence the parameter settings. Moreover, disease models, such as those for atherosclerosis, may suffer from turbulent flow^44^. Intravoxel dephasing may induce significant error in estimates of the peak velocity. Third, to complete the entire scan session, each rat had to be anesthetized for 90 min. Thus, maintaining a stable hemodynamic situation for such long periods of anesthetization could be a major concern. In our experimental design, we counterbalanced the scan orders of varied VENC settings or different resolution parameters across rats. The main effect of depth of anesthesia could be minimized, and the conclusions described above should be valid. Finally, no validations by phantom experiments in small tubes were conducted in this study. The comparisons with phantom measurements are suggested for future work.

In conclusion, we have shown that hemodynamic measurements in rat CCA, such as blood flow, WSS, and artery area, are influenced by the usage of gated/non-gated sequences and the spatial resolution of PC-MRI measurements. However, the effect of different VENC settings is small if the VNR is sufficient. With the scanning parameters suggested in this study (PC-MRI with a gated sequence, VENC = 120 cm/s, and a spatial resolution of 0.21 mm), the corresponding intrascan CoVs were 7.4% and 7.9% for blood flow and WSS measurements, respectively. This suggests PC-MRI can be a useful approach to obtaining flow-related indices in rat CCA in the future.

## Methods

### Animal preparation

All the animal experiments was approved by the China Medical University Institutional Animal Care and Use Committee and were carried out in accordance with the approved guidelines. All MRI examinations were performed on a 7-Tesla animal MRI scanner (ClinScan 70/30, Bruker, Germany) with a gradient strength of 630 mT/m. A total of 24 SD rats (weight: 280–350 g) were scanned in this study. During the scans, the animals were anesthetized with isoflurane through a nose cone. The anesthesia dose was 5% for initial induction and 2% for maintaining the heart rates of rats within the range of 40–50 bpm. The respiratory rate and electrocardiography (ECG) gating signals were monitored simultaneously during scanning.

### Study 1

**Figure 5:**
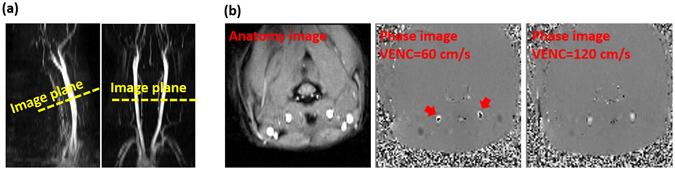
Illustration of the position of PC-MRI scans and representative images. (**a**) Example of PC-MRI slice position on the reconstructed sagittal and coronal view from the time-of-flight (TOF) angiogram. The yellow line indicates the image plane of PC-MRI at the level of middle of common carotid artery (CCA). (**b**) Anatomy and phase images from a representative rat. Red arrows indicate the phase aliasing when VENC of 60 cm/s was used. With higher VENC setting, the phase aliasing vanished.

The purpose of this study was to evaluate the effects of cardiac-gated/non-gated sequences and VENC on blood flow, WSS, and artery area measurements. A total of 12 SD rats were scanned in this study. A 2D time-of-flight (TOF) angiogram was first made to obtain anatomical information on the localization of the CCA. The scanning parameters were TR/TE = 22/4.87 ms, flip angle = 90°, field of view (FOV) = 40 × 40 mm, matrix size = 256 × 256, slice thickness was 0.6 mm, and the NEX = 1. Following localization with the TOF angiogram, the image plane of the PC-MRI was targeted at the middle of CCA and perpendicular to the direction of blood flow (Fig. 5a). Next, both gated and non-gated PC-MRI scans were performed to determine the optimal settings for measuring the relevant hemodynamic parameters. For both gated and non-gated techniques, VENC values in the range of 60–120 cm/s with an interval of 10 cm/s were scanned twice in randomized order to estimate the CoV. The reproducibility experiment for each VENC setting was separated by other VENC settings to avoid measurement bias. Any velocity aliasing (usually at the center of CCAs) due to underestimated VENC parameters was corrected by post-processing^24^. With a VENC of 120 cm/s, no phase aliasing was observed in any rats (Fig. 5b). Other imaging parameters of the 2D time-resolved PC-MRI scans were as follows: TR/TE = 15.55/4.51 ms (minimum TR and TE), flip angle = 30°, FOV = 40 × 40 mm^2^, matrix size = 128 × 128, slice thickness was 2 mm, and the NEX = 8. Unidirectional VENC was done in the through-plane direction. The temporal resolution was around 31 ms. For the cardiac-gated technique, a prospective ECG gating technique was used to synchronize the heartbeat and acquire flow information within 90% of the R–R intervals. Total scan durations were 3.5 mins and 16 s for gated and non-gated sequences, respectively.

Each PC-MRI scan generated three images: an anatomic image, a magnitude image (or named complex difference image), and a velocity image. The phase-offset errors arising from non-compensated eddy-current-induced fields and concomitant gradient terms were first corrected as previously discussed^45^. ROI of the CCA was manually delineated by a rater with 4 years of experience in PC-MRI ROI drawing. The rater carefully drew the ROI on the magnitude image by tracing the boundary of the CCA. The defined ROI were copied to the phase images to compute the blood flow velocity, blood flow volume, WSS, and artery area by an in-house developed program written in Matlab (The MathWorks, Natick, MA, USA). For each time frame image, the vessel lumen was first segmented using cubic B-splines^46, 47^. For a Newtonian and incompressible fluid, the WSS tension ($\vec{\tau }$) can be estimated based on the deformation tensor at the vessel wall:

1 $$\mathop{\to }\limits_{\tau }=2\eta \,\dot{\varepsilon }$$

where η is the viscosity of fluid and $\dot{\varepsilon }\,\,$ is the deformation tensor. Assuming that there exists a 1D problem, the WSS tension can be simplified by the following equation^11^:

2 $${\rm{WSS}}={\rm{\eta }}\frac{d\nu }{dr}$$

where η is assumed as 3.5 × 10^−3^ N s/m2 in a rat model^48^, *v* is the fluid velocity, and r is the vascular radius. For the gated sequence, the WSS was averaged over the cardiac cycle and the circumference of the vessel wall; for the non-gated sequence, the WSS was only averaged in terms of spatial aspect.

The test–retest reproducibility of each sequence with varied VENC settings was assessed by the CoV calculated as following:

3 $$Co{V}_{j}=\frac{1}{I}\,\sum _{i}\,\frac{S{D}_{k}(WS{S}_{ijk})}{mea{n}_{k}(WS{S}_{ijk})}$$

where WSS_ijk_ means the k^th^ repetition of WSS measurement of rat #i (i = 1, 2, 3…, I, I = 12 in this study) with VENC setting j (j = 60, 70, 80, 90, 100, 110, and 120 cm/s). Mean_k_ and SD_k_ respectively symbolize the average and standard deviation of WSS across repetitions. In addition, the CoVs of blood flow and artery area measurements were evaluated.

To compare data between gated and non-gated sequences with varied values of VENC, two-way ANOVA tests with repeated measures were performed on blood flow, WSS, artery area, and their corresponding CoVs. If the effect was observed in the ANOVA analysis, post-hoc Tukey’s HSD test was employed. The data are expressed as mean ± standard error. A *P* < 0.05 was considered statistically significant.

### Study 2

To account for the tradeoff between time and accuracy, the effect of spatial resolution on blood flow, WSS, and artery area measurements was investigated in Study 2. Another 12 SD rats were scanned in this study. As in Study 1, the 2D TOF angiogram with identical parameters was employed in Study 2 for CCA visualization. The image plane of PC-MRI was placed perpendicularly to the middle of CCA.

In-plane resolutions of 0.63, 0.31, 0.21, and 0.16 mm (the corresponding matrix size were respectively 64 × 64, 128 × 128, 192 × 192, and 256 × 256 at a given FOV of 40 × 40 mm^2^) were assessed. The numbers of repetition were respectively 4, 8, 12, and 16 for resolutions of 0.63, 0.31, 0.21, and 0.16 mm. The corresponding scan times for each resolution setting were around 1 min, 3.5 min, 8.5 min, and 13.5 min (the actual scan time may be slightly different among rats due to the varied cardiac cycles), respectively. Note increasing the numbers of repetition yielded a longer scan time. To evaluate the test–retest reproducibility, each resolution was scanned twice to assess the CoV. The scanning order of each resolution was counterbalanced across rats. The other scanning parameters for PC-MRI were as following: TR/TE = 15.55/4.51 ms (minimum TR and TE), flip angle = 30°, and slice thickness = 2 mm. The scanning strategy and VENC setting optimized from Study 1 were applied.

To compare the differences across different resolutions, one-way ANOVA tests with repeated measures were performed on blood flow, WSS, artery area, and their corresponding CoVs. Ideally, the higher imaging resolution was expected to present the more accurate measurements due to less partial voluming effect, and vice versa. Therefore, if the resolution effect was detected in the ANOVA analysis, results from each resolution were compared with a resolution of 0.16 mm by using post-hoc Tukey’s HSD test. The data are expressed as mean ± standard error. A *P* < 0.05 was considered to be statistically significant.

## Electronic supplementary material

Supplementary information
