# Supplementary material for: Optimized analysis of blood flow and wall shear stress in the common carotid artery of rat model by phase-contrast MRI
Source: Sci Rep. 2017 Jul 12;7:5253. doi: 10.1038/s41598-017-05606-4 (PMC5507910; doi:10.1038/s41598-017-05606-4)
Supplement: Supplementary file 1 — Supplementary information [file 41598_2017_5606_MOESM1_ESM.pdf]

Supplementary Information

Optimized analysis of blood flow and wall shear stress in the common carotid artery of rat model by phase-contrast MRI

Shin-Lei Peng,[1*] Cheng-Ting Shih,[2] Chiun-Wei Huang,[3] Shao-Chieh Chiu,[3] and Wu-Chung Shen,[1,4]

[1]Department of Biomedical Imaging and Radiological Science, China Medical University, Taichung, Taiwan; [2] 3D Printing Medical Research Center, China Medical University Hospital, China Medical University, Taichung, Taiwan; [3]Center for Advanced Molecular Imaging and Translation, Chang Gung Memorial Hospital, Taoyuan, Taiwan; [4]Department of Radiology, China Medical University Hospital, Taichung, Taiwan.

*Correspondence to Shin-Lei Peng: speng@mail.cmu.edu.tw

Figure S1.

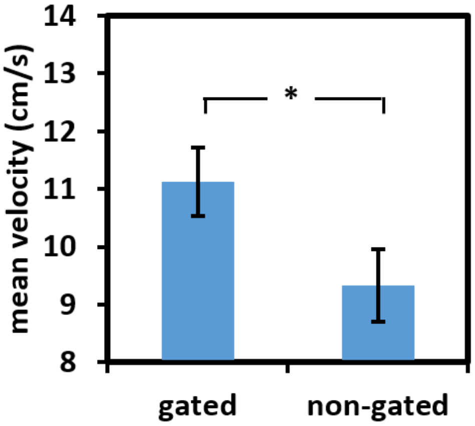

Figure S1: The comparison of mean velocity within CCA between gated and non-gated sequences. The mean velocity measured from the non-gated sequence was significantly lower than that from the gated sequence (*: $P < 0.05$).

Figure S2.

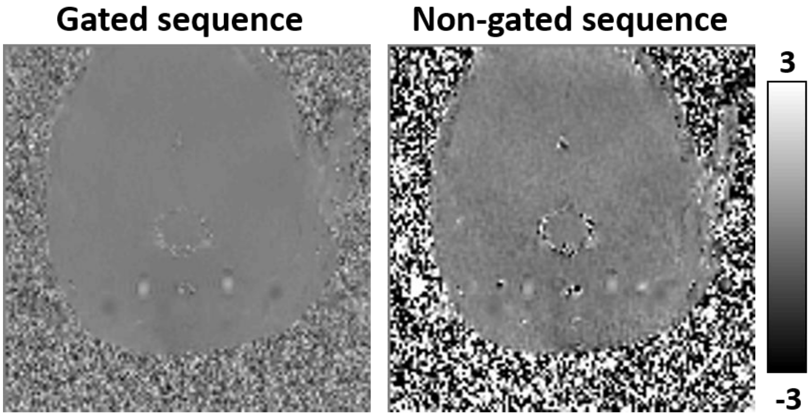

Figure. S2. The relative velocity maps from a representative rat. From the visual inspection, these two relative velocity maps obtained by different sequences have virtually identical image contrast, suggesting the similar flow velocity gradient (Relative velocity = individual velocity/max velocity).
